# Who am I? Self-inquiry as a neurophenomenological approach to consciousness

**DOI:** 10.3389/fpsyg.2026.1951193

**Published:** 2026-09-22

**Authors:** Samhitha Tirumalasetti

**Affiliations:** GITAM Institute of Medical Sciences and Research, GITAM Deemed to be University, Visakhapatnam, India

**Keywords:** Advaita Vedanta, decentering, ego dissolution, meta-awareness, minimal phenomenal experience, minimal self, neurophenomenology, nondual awareness

## Abstract

Inconclusive evidence for leading third-person theories of consciousness has renewed scientific interest in its first-person nature. Self-inquiry, a first-person method from Advaita Vedanta, can be operationalized to meet this need, though it has not received prior empirical study, likely owing to Advaita’s idealism. By bracketing Advaita’s metaphysical commitments and mapping its characterization of consciousness onto the distinctions drawn by consciousness science, self-inquiry can be understood as a systematic phenomenology of selfhood. The method’s rationale is that mental contents perpetuate the “narrative self,” or one’s personal identity, so tracing such contents back to the sense of “I” to which they are referred attenuates the narrative self and discloses the “minimal self.” The minimal self is the pre-reflective first-person perspective of experience that carries a sense of “for-me-ness.” Sustained attention to the minimal self may then facilitate a selfless mode of awareness, or nondual awareness, marked by the absence of felt division between self and world. On this account, the narrative self and the appropriative ownership underlying the minimal self are seen as illusory, while an impersonal, reflexive consciousness remains. This deconstruction of selfhood already has neurophenomenological precedent, though from studies confined to Buddhist practice and reliant on idiosyncratic techniques. Self-inquiry thus holds empirical significance: its stepwise structure offers potential reproducibility across participants, a within-practice control, and a target attentional operation whose proposed distinctiveness can be empirically tested. It may also hold clinical relevance, having been proposed for incorporation into psychotherapy amid growing research into the therapeutic potential of self-attenuation.

## 1 Introduction

By its nature, consciousness is a first-person phenomenon, yet the scientific frameworks developed to explain it are predominantly third-person in orientation (Gallagher and Zahavi, 2020). A recent adversarial collaboration directly testing the two most prominent frameworks—Global Workspace Theory and Integrated Information Theory—proved inconclusive, supporting some predictions while challenging key tenets of both (Cogitate Consortium et al., 2025). At the same time, recent work emphasizes the need for integration of the first-person perspective (Schmidt et al., 2024). As Cleeremans et al. (2025) note, “consciousness research may benefit from a stronger focus on the phenomenological and experiential aspects of conscious experience.”

Neurophenomenology, pioneered by Varela (1996), proposes that research should proceed through systematic coordination between first-person phenomenological accounts and third-person neuroscientific data, operating as mutual constraints rather than as independent parallel tracks. Carefully trained phenomenological descriptions can provide rigorous first-person data capable of constraining the interpretation of neural findings, while those findings in turn refine and sharpen phenomenological descriptions. Neither side is treated as ground truth; each is iteratively refined in light of the other.

Varela conceived neurophenomenology to address a specific challenge: the “hard problem of consciousness,” which concerns how and why physical processes in the brain give rise to subjective experience (Chalmers, 1995). Rudrauf et al. (2003) characterize Varela’s methodology as rejecting eliminativist approaches that dismiss subjective experience as a genuine scientific explanandum, while also refraining from metaphysical positions that treat consciousness as ontologically primary. Instead, they advocate for an empirical agnosticism in which the relationship between subjective experience and neural dynamics remains an open question to be investigated rather than presupposed.

This stance motivates an agnostic operationalization of self-inquiry, a first-person method from Advaita Vedanta (Maharshi, 2010; Dahl et al., 2015)—a tradition that holds consciousness to be ontologically primary (Juturi, 2020). To the author’s knowledge, self-inquiry has been extensively elaborated in philosophical literature (Maharshi, 2000; Davis, 2010; Maharshi, 2010; Godman, 2019; Tripathi, 2025) but has received virtually no direct empirical study. Where it appears in the contemplative neuroscience literature, it is named in passing among other practices (Dahl et al., 2015; Ramírez-Barrantes et al., 2019), though a recent account offers a cognitive characterization of the practice in its own right (Maleeh and Konjedi, 2026). This gap exists despite a recent call for its incorporation into psychotherapy (Zhao, 2025) and growing scientific interest in “nondual awareness” more broadly (Josipovic, 2014; Josipovic, 2019; Meling, 2022). Because self-inquiry is embedded within Advaita’s metaphysical and devotional language (Maharshi, 2010), it may have remained difficult to investigate empirically. The present paper thus brackets the tradition’s metaphysical commitments while reformulating self-inquiry’s phenomenological observations in scientifically accessible terms.

The central claim of the paper is that self-inquiry is tractable for neurophenomenological investigation: its stepwise structure (Maharshi, 2010) isolates a distinct attentional operation that may be temporally located via neural measurement, reported upon in the first person, and compared against a within-practice control. Its standardization may also render it reproducible across participants and sessions. The paper first develops a conceptual account of self-inquiry and then establishes its empirical tractability.

## 2 Advaita Vedanta: ontology and self-inquiry

To situate self-inquiry, its tradition’s account of consciousness must first be outlined—not as a set of claims the present paper endorses, but as the conceptual framework from which the method emerges.

Advaita Vedanta is a school of Indian philosophy that originates in the Upanishads of the first millennium BCE and was later systematized by Adi Sankaracharya in the eighth century CE (Navone, 1956; Sarker, 2021). It characterizes consciousness as the fundamental reality underlying all existence. Within this tradition, the universe is understood as appearing within consciousness rather than existing independently of it. Consciousness is therefore not treated as a property of the brain; instead, the brain itself—along with all other matter—is taken to arise within consciousness (Juturi, 2020).

Multiplicity is correspondingly regarded within this tradition as an illusion (*maya*) rather than as ultimately real (Sarker, 2021). This is often illustrated through the analogy of dreaming: in a dream, the dream-self, other characters, and the entire environment all arise from and appear within a single source, despite presenting themselves as separate entities (Maharshi, 2010; Dalal, 2022).

Self-inquiry is understood within Advaita Vedanta as a method (Dahl et al., 2015) for “awakening” from *maya* and thereby recognizing the non-independent status of appearances (Maharshi, 2010; Sarker, 2021).

## 3 Conceptual mapping: Advaita Vedanta and consciousness science

The Advaitic view can also be expressed schematically. Swami Sarvapriyananda, Resident Minister of the Vedanta Society of New York, offers an accessible equation: Consciousness + Object = Experience (Balasubramanian, 2011; Fasching, 2021; South Park Commons, 2026). For the purposes of the present analysis, the relation may be more precisely rendered as Consciousness → Object = Experience. On the Advaitic view, “Object” arises from “Consciousness” rather than existing independently of it, so the arrow notation makes this ontological dependence explicit (Maharshi, 2010; Juturi, 2020; Dalal, 2022). In its scientific usage, the term “consciousness” refers to several distinct concepts (Cleeremans et al., 2025)—a number of which map onto the “Object” term of this Advaitic formula, but not onto the first term, “Consciousness” in the Advaitic sense.

In consciousness science, “perceptual awareness” refers to the awareness of information given through the senses, whether of the external environment or of the body’s states and position within it (Deroy et al., 2016; Cleeremans et al., 2025). “Contents of consciousness” is broader, referring to any item present in experience at a given moment, including thoughts, perceptions, memories, and emotions (Halligan and Oakley, 2021; Cleeremans et al., 2025). Both describe consciousness *of* something—a feature of experience that philosophers call intentionality (Cleeremans et al., 2025). Ordinarily, experience is described as having two poles: a subject to whom something appears, and an object that appears. Intentionality is the relation between the two poles (Gupta, 2004), such that to be conscious requires being conscious of something (Cleeremans et al., 2025). Since Consciousness in the Advaitic sense is ontologically primary, it “is not intrinsically object-directed or intentional” (Gupta, 2004). In other words, it does not depend on any relationship with objects to exist (Juturi, 2020), which the arrow notation adopted earlier reflects. Thus, the terms “perceptual awareness” and “contents of consciousness” fall on the side of Object—they concern what appears within experience (Maharshi, 2010).

“Self-awareness,” by contrast, refers to the subject pole of experience. It is typically defined as the experience of oneself as distinct from the environment, involving a sense of an “I” that has a story and persists over time (Morin, 2011). Phenomenology further distinguishes self-awareness into two modes: the narrative self and the minimal self (Dor-Ziderman et al., 2013). The narrative self refers to one’s personal identity; selfhood emerges from the organization of one’s deeds and events across life into a unified picture (Čapek, 2017). On the other hand, the minimal self refers to the first-person perspective of experience, which carries a sense of “for-me-ness”: the sense that experience is always given to a point of view. The minimal self is pre-reflective, meaning that it is present without requiring one to explicitly take note of it (Zahavi and Kriegel, 2016). In Metzinger’s self-model theory, the self is a construction of the brain, so “for-me-ness” would, on this view, be a feature of that construction rather than amounting to a self. Metzinger argues that schizophrenic patients who report thoughts as inserted, and not their own, demonstrate that “for-me-ness” is not necessary for experience (Metzinger, 2003). However, Zahavi argues that such thoughts are still registered by the patients; what is denied is authorship of the thoughts rather than their givenness (Zahavi and Kriegel, 2016).

Advaita occupies an intermediate position between Metzinger’s self-model theory and Zahavi’s phenomenological account of the minimal self. It converges with Metzinger in regarding the self as illusory but with Zahavi in affirming the enduring existence of a reflexive consciousness—one that is present to itself, yet not belonging to an “I” (Ram-Prasad, 2010). In self-inquiry, the “I” is regarded as the primary content to which all others are implicitly referred. Thoughts and perceptions are taken to belong to an “I”: they are my thoughts, my body, and so on. For this reason, self-inquiry takes the “I”-sense—the sense that experience, in being given, is given *to* a self—as its specific target, treating it as the anchor of misidentification (Maharshi, 2010). What it works to dissolve is this appropriative ownership rather than “for-me-ness” as such (Ram-Prasad, 2010); prior work has found precisely this ownership layer of the minimal self to be attenuable, with a residual mode of “selfless” experiencing remaining (Dor-Ziderman et al., 2013). Thus, self-inquiry directs sustained attention toward this “I,” the felt sense of subjectivity, rather than analyzing it conceptually. On the tradition’s account, when attention remains fixed in this manner, the personal sense of subjectivity is said to gradually dissolve altogether (Maharshi, 2010). What is said to remain, once the “I”-sense no longer obscures it, is Consciousness in the Advaitic sense (Maharshi, 2010; Sarker, 2021), described in the philosophical literature as “witness-consciousness” (Albahari, 2009).

Metzinger has approached this same witness-consciousness under a different name—“minimal phenomenal experience,” or the felt quality of “pure awareness” in meditation. He theorizes this state to be a predictive representation of tonic alertness, meaning that the brain models its own baseline wakefulness (Metzinger, 2020). The MPE-92 M questionnaire was then developed as a phenomenological self-report instrument to profile the state (Gamma and Metzinger, 2021). Like the present treatment, it brackets the metaphysics to isolate the phenomenology. It is, however, directed at characterizing the state itself (Metzinger, 2020); the present concern is a method by which that state may be approached—a complementary undertaking.

The rationale for self-inquiry rests on a distinction between observer and observed (Singh, 2022). Thoughts and memories can be observed in contemplative practice, and the body can likewise be perceived (Juturi, 2020; Sarker, 2021). Even one’s personal sense of subjectivity, what self-inquiry refers to as the “I”-sense, can be apprehended (Maharshi, 2010). By contrast, the observer itself is said to resist objectification (Gupta, 2004; Preti, 2011; Juturi, 2020; Fasching, 2021). In the philosophical literature, witness-consciousness is described as unable to directly observe itself yet as remaining intrinsically aware of its own existence (Albahari, 2009). Therefore, “self-awareness,” insofar as it involves a sense of an “I,” falls on the side of the Object: another content of consciousness that is ordinarily, though mistakenly, identified as the real subject (Balasubramanian, 2011).

By the same reasoning, the mind–body self, or the sense of self as it relates to being a psychological or physical entity, pertains to the Object as well (Juturi, 2020; Sarker, 2021). Thus, concepts such as “levels of consciousness” and “functional (or access) consciousness” also fall outside the first Advaitic term, as they describe states of arousal or cognitive functions (Cleeremans et al., 2025).

One scientific concept, however, resists this sorting altogether by not mapping onto any single term of the Advaitic formula; rather, it concerns the formula as a whole. “Phenomenal consciousness”—the subjective, qualitative character of experience—is the focus of the hard problem of consciousness (Chalmers, 1995; Cleeremans et al., 2025). In the standard physicalist framing, the problem derives its difficulty from beginning with the Object—the brain and physical processes—and then having to explain how Experience could arise from matter alone. On the Advaitic view, this difficulty is an artifact of the starting point. The tradition begins with Consciousness as ontologically primary, so the question does not arise as an explanatory problem (Juturi, 2020).

Beyond its metaphysics or its characterization as witness-consciousness and minimal phenomenal experience, Advaita’s designation of Consciousness corresponds closely to what a growing body of work terms nondual awareness (Josipovic, 2014; Josipovic, 2019). A clarification is needed here: nondual awareness, as a target of study, is a recognizable state whose neural correlates are being investigated and hypothesized (Josipovic, 2014; Josipovic, 2019). Consciousness in the Advaitic sense—the fundamental nature of reality—is metaphysical and therefore not measurable (Preti, 2011; Juturi, 2020; Fasching, 2021). To investigate the correlates of nondual awareness is thus to investigate a person’s recognition of nonduality—the absence of a felt division between self and other, or between self and world (Mills et al., 2020)—not to measure the metaphysical claim itself.

The nature of nondual awareness is debated: Albahari holds that the constructed self must dissolve for nondual awareness to be recognized (Albahari, 2024), whereas Josipovic argues that it can be recognized while the sense of self remains intact (Josipovic, 2025). This paper suggests that the two positions can be reconciled if they are understood to describe nondual awareness at different timescales: for Josipovic, a state, entered and exited as the sense of self is suspended and returns; for Albahari, a trait, the abiding realization in which the felt “I” has fallen away for good. This distinction is relevant because self-inquiry may apply to both.

## 4 Self-inquiry as a first-person method

Advaita Vedanta does not merely make a metaphysical claim; it proposes a method of investigation (Dahl et al., 2015)—what might, in modern terms, be described as a systematic phenomenology of selfhood. While the inferential move from phenomenological observation to metaphysical conclusion is contestable, the method itself can be evaluated independently of that move. Ramana Maharshi, widely regarded as the leading proponent of experiential Advaita (Sharma, 1993), articulated instructions in the twentieth century that remain amenable to operationalization (Maharshi, 2010). Notably, he held that self-inquiry is to be verified through direct personal investigation rather than accepted on doctrinal authority, thereby locating epistemic authority with the practitioner (Davis, 2010). His method proceeds as follows (Maharshi, 2010):

When mental contents (e.g., thoughts, emotions, or memories) arise:

1. Notice them; do not pursue them.
2. Ask: “To whom do they arise?”
3. Answer: “To me.”
4. Inquire: “Who am I?”
5. Hold onto the sense of “I”—in other words, sustain attention on the felt sense of subjectivity.
6. Repeat each time the mind wanders.

Experienced practitioners report that Steps 2–4 may be omitted once sufficient familiarity is established (Godman, 2019).

One feature of the method warrants clarification. The question “Who am I?” can become an intellectual inquiry—a matter of conceptual reflection—which alters its intended function. The aim is not to arrive at a propositional answer but to redirect attention toward the felt sense of “I” (Godman, 2019). At first glance, self-inquiry may appear inherently propositional, since “Who am I?” is a question bound to language and concept. Ramana Maharshi explained, however, that although the question is itself a thought, it functions like “a stick used for stirring the burning pyre”—a means that ultimately becomes unnecessary once its function has been fulfilled (Maharshi, 2010).

With this clarified, the operative step can be identified: holding onto the sense of “I.” Step 5 is of central interest, both within the tradition (Maharshi, 2010; Godman, 2019) and for future empirical investigation. It also illuminates the structural logic of self-inquiry. Practices that attend to the mind’s contents are regarded as facing an open-ended task: contents are innumerable and ceaselessly arising, so there is always another to notice. Self-inquiry, by contrast, focuses on a single target—the “I”-sense to which these contents are referred—treating it as the root to be addressed. On this view, many forms of meditation remain directed toward such contents rather than the source to which they are attributed (Maharshi, 2010).

## 5 Self-inquiry in relation to other contemplative practices

Several contemplative practices resemble self-inquiry (Dahl et al., 2015; Ramírez-Barrantes et al., 2019), making it useful to clarify what makes self-inquiry distinct.

Husserl’s phenomenological reduction (*epoché*) brackets assumptions about the existence of the world; in other words, the reality or unreality of objects is not relevant to the method. Rather, the aim is to examine how objects present themselves to consciousness. Through this reduction, Husserl discloses what he calls the “transcendental ego,” distinct from the “empirical ego” of everyday experience. Whereas the empirical ego refers to the psychological person, the transcendental ego is the “I” as the subject for whom objects appear in experience (Gupta, 2004). Self-inquiry begins from a related observation: every experience belongs to an “I” (Maharshi, 2010). Unlike Husserl’s reduction, however, self-inquiry proceeds to investigate this “I” in order to dissolve it and disclose what Advaita regards as a fundamental, impersonal consciousness (Gupta, 2004; Maharshi, 2010).

A related approach can be found in Early Buddhism—a term referring to the original teachings of Gautama Buddha rather than the later schools that developed from them. The teaching of *anatta*, or not-self, refers to the absence of a permanent, unchanging self. Selfhood is not denied outright, but is understood as the product of “a rapidly shifting stream of momentary mental occurrences” (Hoang, 2019). Thus, *anatta* investigation systematically examines the contents of consciousness—body, sensations, thoughts—observing each to be impermanent and therefore not a stable self (Hoang, 2019). Advaita Vedanta has a comparable practice, *neti neti* (“not this, not that”), in which such contents are similarly rejected as not-self. However, both *anatta* investigation and *neti neti* proceed by examining each content in turn rather than directly investigating the self to which these contents are referred. Self-inquiry, according to Maharshi, turns directly to the root of selfhood, the felt “I”-sense that appropriates consciousness, in order to dissolve it (Om, 1981; Maharshi, 2000; Das, 2020).

A closer parallel to self-inquiry is found in Mahamudra, an esoteric tradition within the later schools of Indian and Tibetan Buddhism. Mahamudra comprises several distinct practices for cultivating nondual awareness, not all of which are available to laypeople. Insight meditation, however, is one such approach. The practitioner “first examines the mind in movement and at rest” and then “cuts to the root” of mind to discover that it has no intrinsic nature, being ultimately “empty, luminous, non-dual, and blissful.” Because this process can be difficult to grasp, the tradition relies on “pointing-out instructions” from a guru, or qualified teacher, to clarify it for the practitioner (Jackson, 2011). Within Advaita Vedanta, Ramana Maharshi is regarded as such a teacher (Sharma, 1993), and his instructions on how to engage in a similar process are precise. For instance, examining the mind in movement can be understood to correspond to the first step of self-inquiry, in which a practitioner notices mental contents as they arise. The “root” of mind, as per Maharshi, is the “I” to which such mental contents belong. Step 5, the one of central importance to the method, entails sustained attention to the “I”-sense, or felt sense of subjectivity. According to Maharshi, the sense of self dissolves under such scrutiny to reveal nondual awareness (Maharshi, 2010). Where less specific instructions may leave the root underdetermined without a teacher’s guidance, Maharshi’s recorded explanation of self-inquiry names the felt “I”—the ownership layer of the minimal self—as its explicit target.

Notably, Mahamudra and Advaita have been explicitly named in a recent account noting the relative scarcity of empirical research on nondual practices. The account proposes that a nondual shift requires two components—non-adaptivity (non-judgment) and non-propositional meta-awareness (self-knowing awareness)—and then evaluates these against Mahamudra. It remains, however, a theoretical account: it identifies what a nondual shift may require and explicitly calls for the future operationalization of its proposed components (Meling, 2022).

Self-inquiry is well positioned to meet this broader interest. Because Ramana Maharshi articulated it as a relatively fixed, stepwise procedure, it allows a discrete, reproducible operation to be isolated—holding attention onto the “I”-sense—at which potential neurophenomenological changes can be observed. The following section develops this possibility.

## 6 Empirical tractability

The deconstruction of selfhood described by Advaita Vedanta already has empirical precedent. Dor-Ziderman et al. (2013) conducted a neurophenomenological investigation in which MEG recorded the neural correlates accompanying the attenuation of both the narrative and minimal self, along with the resulting experience of selflessness. However, the study was conducted with 12 Buddhist practitioners averaging 11,225 h of meditation across 16.5 years. The authors themselves acknowledge this limitation—the small sample and lack of generalizability—noting that their results “warrant replication in a larger group and in other meditative traditions.”

Subsequent work has extended such investigations to larger samples (N = 46), though these remain within Buddhist practice and rely on individually chosen techniques to produce self-boundary dissolution (Nave et al., 2021; Trautwein et al., 2024). This highlights the need for a standardized contemplative method from a distinct tradition—a role self-inquiry may fulfill. Because it is taught as a stepwise procedure, it has the potential to be uniform across participants and reproducible across sessions—features that make it tractable for research.

Self-inquiry also aligns with recent neurophenomenological work that shifts focus from what is experienced to how (Sandved-Smith et al., 2025). Its procedure is explicitly reasoned by Maharshi (2010):

1. Mental contents perpetuate the narrative self.
2. Tracing such contents back to the “I”-sense to which they are referred attenuates the narrative self and discloses the minimal self.
3. Sustained attention to the minimal self can then facilitate a selfless mode of awareness.

The practice thus supplies a defined sequence of steps rather than a target state alone. Recent MEG work has focused on the target state—the dissolution of self-boundaries (Trautwein et al., 2024)—reached through various participant-selected gestures of “letting go” and relaxation rather than a standardized procedure (Nave et al., 2021). Self-inquiry differs by specifying a single directed operation—holding attention onto the felt “I”—thereby rendering it available for neural measurement. Prior work has already detailed how to implement neurophenomenology in such contexts (Berkovich-Ohana et al., 2020), while micro-phenomenological interviewing (a second-person method) additionally offers an established means of eliciting fine-grained experiential descriptions (Petitmengin, 2006; Hruby et al., 2026). Thus, the aim of the present paper is to establish self-inquiry’s tractability; designing a specific experimental paradigm remains a task for future work.

The stepwise structure of self-inquiry offers a further methodological advantage: a within-practice control. The target operation—holding attention onto the felt “I”—can be compared against the first step in the same practice: noticing one’s thoughts, which plausibly engages the kind of meta-awareness modeled computationally in studies of mental action (Sandved-Smith et al., 2021). Both are acts of attention directed toward an object—a mental content in one case versus the felt “I” in the other—so the difference between them may be attributed to the target operation itself rather than to a change in the type of processing involved. If the target operation proves neurophenomenologically distinguishable from its control, the hypothesis that it constitutes a distinct attentional operation is supported; a null result would weigh against it, though only where successful enactment can be established.

Because the target and control steps are performed by the same participant, this comparison avoids the between-group confounds common to contemplative neuroscience. It also offers an advantage over existing active control conditions. Since comparisons against rest are nonspecific for meditation, a prior study contrasted self-boundary dissolution with a condition of maintaining self-boundaries (Trautwein et al., 2024). However, dissolution was reached through idiosyncratic techniques and maintenance through focused bodily attention (Nave et al., 2021), so the conditions differed in several respects at once. The comparison proposed here varies only in the object of attention.

Nevertheless, this comparison has limitations: the two steps differ in duration and temporal position, so a specific paradigm would need to account for order effects such as drift or fatigue. They may also differ in difficulty: sustaining attention on the felt “I” is more demanding than noticing a thought, and a mental content, once noticed, cannot be dwelt upon without elaboration—which the first step excludes. A paradigm would need to account for these asymmetries and to verify that participants successfully enacted each step—for which retrospective success and stability ratings, used to rule out task-difficulty confounds across conditions, offer an established precedent (Dor-Ziderman et al., 2013). The intermediate steps (Steps 2–4) are not suitable as controls, being propositional in nature. They may, however, scaffold participants naive to self-inquiry, though not necessarily to contemplative practice (Godman, 2019). More broadly, the method remains contingent on participants’ capacity for introspection, which may constrain its application and interpretation. How much prior contemplative experience this capacity requires is undetermined, though existing work has included practitioners with as little as roughly 115 h (Nave et al., 2021), suggesting the necessary baseline need not be extensive.

A final limitation is contextual: self-inquiry is a practice intended for daily life and is not a formal seated exercise (Maharshi, 2000), yet any laboratory implementation necessarily removes it from that native context. Daily activity continually provides occasions to intercept arising mental content and trace attention back to the “I,” but a laboratory setting may not facilitate this natural stream of consciousness. In this setting, a participant with extensive contemplative experience may fail to produce the spontaneous thoughts on which the control step depends—a risk any implementation would need to account for. Findings would therefore speak to the operation as performed in the laboratory, which may differ from its enactment in daily life. Taken together, these constraints imply a specific target population: participants with some contemplative background but naive to self-inquiry, capable of sustained introspection, and able to generate the spontaneous mental content the control step depends on.

## 7 Clinical implications

Contemplative methods may not only serve as psychotherapeutic interventions but also as a means for developing computational neurophenomenology capable of generating therapeutic predictions. A mindfulness-based intervention, for instance, is simultaneously a way of training a patient to accurately report on their experience and a treatment that relieves suffering. Such an intervention thus imparts both epistemic and therapeutic gains, where each constrains the other. Refined experiential description can sharpen a clinically informed model, the model then identifies a suitable intervention, and the intervention in turn yields further phenomenological data. This approach has been proposed as a response to the difficulty of specifying how psychiatric treatments actually work (Lutz et al., 2025).

This cyclical process is dependent on the epistemic capacities honed by the contemplative method used. For example, one of the epistemic outcomes seen in mindfulness-based cognitive therapy is increased meta-awareness (Lutz et al., 2025), in which a patient has heightened awareness of mental processes (Lutz et al., 2015). However, this outcome is not necessarily beneficial on its own. A hypothetical scenario of a patient with anxiety can exemplify this: if such a patient perceives an elevated heart rate, the awareness itself might trigger further anxiety, since the sensation is still taken to signal genuine threat. Thus, what is additionally required is dereification or “decentering,” in which the patient no longer experiences mental processes as accurate depictions of reality (Lutz et al., 2015). In fact, research assessing cognitive therapy and mindfulness-based cognitive therapy as treatment modalities for depression found that both instill the capacity for decentering. It is the continued use of this skill which then predicts relapse prophylaxis independent of residual symptom management (Segal et al., 2019). As self-inquiry is meant for implementation in the midst of daily life rather than as a scheduled formal practice (Maharshi, 2000), it may be well suited for accommodating such continued use.

While decentering modifies the relation between the patient and the contents of awareness (Lutz et al., 2015), the self to which those contents are referred remains presupposed throughout. Thus, a computational model built from phenomenological reports of patients trained in cognitive therapy and mindfulness-based cognitive therapy is constrained accordingly. While such patients may learn to notice mental contents and loosen their grip, the description of experience can only remain at that level. It cannot capture the sense of self or its potential attenuation because the training does not accommodate it. Self-inquiry, by contrast, may not only instill meta-awareness by having the practitioner notice thoughts (Step 1), but it may also encourage decentering by having them ask to whom the thoughts arise (Step 2). In doing so, self-inquiry intends to distance the person not merely from thoughts, as other practices do, but from the thinker itself. It proceeds by having them inquire “Who am I?” (Step 4) and sustain attention on the felt sense of “I” or subjectivity (Step 5), which purportedly facilitates its gradual attenuation (Maharshi, 2010).

A partial exception is acceptance and commitment therapy, which teaches patients to notice an observing self distinct from the thoughts observed. It then has patients use this stance as a self-regulatory strategy when difficult content arises (Hayes and Pierson, 2005). Self-inquiry points at the same observing self but instructs the practitioner to hold attention onto it, eventually loosening ownership over its observational capacity to facilitate a selfless mode of awareness (Maharshi, 2010).

Hence, any psychotherapeutic adaptation of self-inquiry must retain fidelity to its experiential character. While one proposal has explored incorporating self-inquiry into psychotherapy (Zhao, 2025), it risks remaining at a conceptual entry point without reaching the experiential shift on which the practice depends. Although the proposal recognizes that self-inquiry aims beyond cognitive restructuring, its clinical prompts remain largely reflective. For instance, one prompt invites the patient to “reflect on what truly defines you” as the body ages, the mind adapts, and life roles evolve. While such prompts gesture at the instability of the self, they may engage only intellectual reflection—leaving the patient short of experiential self-deconstruction. The tradition itself cautions that self-inquiry must yield experiential rather than merely intellectual knowledge (Maharshi, 2000; Maharshi, 2010).

The experiential knowledge in question is that of self-dissolution (Maharshi, 2010), a phenomenon increasingly examined as a potential contributor to therapeutic outcomes in psychedelic research, particularly in treatment-resistant depression (Stoliker et al., 2022). However, the quality of dissolution plays a key role: positively experienced mystical-type dissolution predicts stronger therapeutic outcomes than dread-laden or anxious dissolution (Roseman et al., 2018)—a distinction corroborated by neural findings (Mason et al., 2020). Related alterations in self-experience also occur in depersonalization and derealization, where they are involuntary and distressing rather than deliberately cultivated (Černis et al., 2025). This contrast highlights the importance of the manner in which attenuation is approached. If self-inquiry is applicable to therapeutic self-attenuation, its gradually progressive nature and independence from pharmacological induction offer potential advantages. Given that the method stands to inform both clinical practice and consciousness science more broadly, the empirical investigation of self-inquiry is warranted.

## Data Availability

The original contributions presented in the study are included in the article/supplementary material, further inquiries can be directed to the corresponding author.
